# Co‐Existence of Divergent Oral Care Approaches in Danish Aged Care: A Qualitative Study

**DOI:** 10.1111/ger.70041

**Published:** 2026-01-05

**Authors:** Linnea Eisemann de Almeida, Louise Folker, Christina Andersen, Astrid Pernille Jespersen, Esben Boeskov Øzhayat

**Affiliations:** ^1^ Research Section for Oral Health, Society, and Technology, Department of Odontology, Faculty of Health and Medical Sciences University of Copenhagen Denmark; ^2^ Copenhagen Centre for Health Research in the Humanities (CoRe), Saxo Institute, Faculty of Humanities University of Copenhagen Denmark

**Keywords:** aged care, dental care, interdisciplinary collaboration, older adults, oral health, qualitative study

## Abstract

**Introduction:**

Care approaches to oral health are shaped by the perspectives of both aged care and dental care professionals. This study provides a new and comprehensive understanding of how these two professional groups conceptualise and address oral care for care‐dependent older adults.

**Material and Methods:**

Researchers conducted 13 focus group discussions with 4–6 participants each in two Danish municipalities within the Lifelong Oral Health research project: nine with aged care staff and four with managers from aged care and dental care. Two semi‐structured interviews were conducted with dental care staff. The research was guided by a qualitative methodological framework using inductive thematic analysis to explore participants' experiences and perspectives.

**Results:**

Aged care and dental care staff held varying perspectives on four distinct care approaches: (1) knowledge of oral health; (2) prioritisation of oral health; (3) care practice in relation to oral health; (4) handling refusal of oral care. In contrast to dental care staff, aged care staff had rudimentary oral health knowledge and prioritised other care tasks over oral care. Aged care staff took a rehabilitative approach to oral care, whereas dental care staff favoured a compensatory approach. Aged care staff stated that the right to self‐determination overruled the duty of care presented by dental care staff when patients refused oral care. These differences affected daily oral care practices and collaboration between both staff groups, who both endorsed better collaboration.

**Conclusion:**

Divergent care approaches to oral care between aged care and dental care staff can hinder good oral health in care‐dependent older adults. To enhance oral health in older adults, this article identifies four divergent approaches and explores how they challenge inter‐professional collaboration.

## 1 Introduction

Reflecting global trends, Denmark's older adult population is expected to increase significantly in coming years [1]. Older adults often become vulnerable due to multimorbidity, substantial medication use associated with polypharmacy and decline in physical and cognitive function [1], leading to an increased demand for care and support. In addition, older adults today retain more teeth than those of earlier generations [1, 2], but they often have extensive repairs, such as fillings, crowns and bridgework; more implants are also seen [3]. This requires sufficient daily oral hygiene practice, but older adults' vulnerable position presents challenges to this [4], and many require help managing their oral care. The consequences of poor oral health in care‐dependent older adults are severe and include toothache, weight loss, malnutrition, weakened general health, worsened quality of life, social isolation and increased mortality [1, 3, 5].

Aged care staff are crucial in providing oral care for older adults [3, 6, 7]. Unfortunately, many older adults living in nursing homes or receiving home care have poor oral health, reflecting challenges in daily oral care practice [3, 6, 7]. Studies suggest that aged care staff lack adequate knowledge of oral health and that a significant discrepancy exists between the actual need for oral care assistance and the service provided by aged care staff [8]. Many initiatives have been launched to sharpen the focus on oral health in the care of older adults, but they have not yielded long‐lasting effects [9], which has been attributed to a lack of willingness among aged care staff and challenges in collaboration between aged care staff and dental care staff [10]. From the latter's point of view, the collaboration is challenged due to perceived ignorance among aged care staff regarding the significance of oral care [11]. By contrast, aged care staff find the collaboration challenging due to a perceived lack of professional acknowledgement from dental care staff, who they believe do not understand the holistic approach needed in caring for older adults [12].

Consequently, solutions are proposed to create structures and processes that support knowledge sharing and creating relationships across the professions [12]. Interdisciplinary health teams have been promoted to avoid professional siloes and make the professions less perplexing to each other [11], which aligns with the notion that interdisciplinary collaboration is crucial in contemporary aged care as promoted by the World Health Organization (WHO) [13]. Successfully coordinating such collaboration will ensure safe, high‐quality care [14].

To engage in interdisciplinary collaborations, it is essential to understand in detail the approaches, views, experiences, challenges and possibilities for oral care in the professions involved, so the present study explored the different approaches to oral care and the existing collaborative practices between aged care and dental care staff. Both aged care and dental care staff can use the findings to enhance collaboration, reduce conflicts and ultimately improve oral health in care‐dependent older adults.

### 1.1 Setting of the Aged Care and Dental Care

The study originated in the Lifelong Oral Health co‐creation research project, which seeks to develop and implement preventive initiatives for improving oral health in care‐dependent older adults. The project is a collaboration between the University of Copenhagen and two Danish municipalities within the capital region. One municipality is densely populated, mainly with young and older adults, and features high average incomes. The other is more suburban, primarily housing families and older adults, with greater socioeconomic diversity. Five of the municipalities' aged care units were involved: two nursing homes, two home care units and a rehabilitation centre. In one municipality, dental care for individuals with reduced physical or cognitive functioning was provided by the publicly administered service, while in the other municipality, the same service was offered by a private company. Older adults living in nursing homes automatically meet the requirements. For others, eligibility for the service is determined through an individual assessment [15].

## 2 Methods

The study adheres to the COREQ checklist [16], which is included as Appendix S1. Data were obtained from 13 focus group discussions and two semi‐structured interviews. Focus groups were chosen to foster an environment in which participants, by drawing on their expertise in their respective fields, could steer the discussion in new directions that the moderator could not anticipate in advance [17]. The moderator followed a topic guide with themes and activities, keeping the format open. The themes included in the topic guide for aged care staff were: (1) the daily care of older adults and (2) daily oral care of older adults. To explore these areas in depth while ensuring participants did not feel exposed or singled out, the guide included exercises designed to elicit participants' understanding of what constitutes good care, concrete experiences of providing effective oral care, and case‐based exercises addressing more challenging aspects of oral care. For managers and dental care staff, themes focused on the organisation, responsibilities, and referral processes for oral conditions and issues. Specific themes addressed were: (1) prioritisation and (2) collaboration between private dentists and municipal services. In addition, case‐based exercises were included to explore the older adult's points of contact within health services, the distribution of responsibilities and the identification of optimal solutions for care provision.

To foster an honest and safe environment, aged care managers and staff were segregated into different groups to prevent a power imbalance. In the focus groups at the management level, managers from aged care and dental care were grouped together. The two semi‐structured interviews were conducted with dental care staff. To avoid group dynamics that might inhibit staff from speaking freely and earnestly, the two staff groups were divided into focus groups and interviews, respectively. Both interviews were conducted as dual interviews with dental care staff, allowing all participants to share their perspectives in a more comfortable and supportive setting. Interviews were chosen for the dental care staff due to the smaller number of participants. All focus groups and interviews were audio‐recorded, transcribed and later translated into English for this article. Each focus group lasted 2 h, and the interviews lasted one and a half hours. The municipalities facilitated the purposive recruitment of managers, aged care staff and dental care staff. Two staff members from the administrative department within the municipalities were responsible for recruiting, booking meeting locations and ensuring provision. The research team provided them with information concerning the proposed group composition, including the number of participants, their job designations and employment details. The admin staff members worked daily outside the care units, maintaining a neutral position regarding specific care staff members. This method facilitated recruitment independent of personal relationships or other internal workplace dynamics. A planned sample size was determined pragmatically based on scope and feasibility. In line with reflexive thematic analysis, we did not aim for data saturation; instead, we sought sufficient richness and diversity to support a meaningful, interpretative analysis. Table 1 shows the composition of the participants.

**TABLE 1:** Overview of participants.

	Job designation	Employment	No. of participants
Aged care	Nursing assistant and trainee, nurse, occupational therapist, educationalist, manager, preadmission evaluator, training coordinator, dementia coordinator	Home care, nursing home, rehabilitation unit	66
Dental care	Dentist, dental hygienist, dental assistant	General practice, privately administered dental care and publicly administered dental care tailored to older adults	14

The research team consisted of ethnologists and odontologists. Both disciplines were represented in designing and facilitating the focus groups, as well as in the analytical process and interpretation of the findings to ensure the incorporation of interdisciplinary perspectives throughout the various phases. Reflexive field notes were written, and a debriefing meeting was held after each focus group to evaluate the session and consider whether any adjustments were needed for subsequent focus groups [18]. Additionally, immediate analytical impressions were discussed, noting surprises, repetitions or other observations that emerged from the focus group.

### 2.1 Reflexivity Statement

The study incorporated both insider and outsider perspectives: the insider perspective of odontologists, as experts in oral health, and the outsider perspective of ethnologists [19]. While the research team was largely composed of outsiders to the aged care setting, some researchers had personal experiences as relatives to older adults receiving care, and one occasionally worked shifts in a nursing home. It is important to note that these personal connections did not involve the aged care settings included in the study. The research team was mindful of these potential biases and engaged in ongoing reflection to address them. The collaboration between odontologists and ethnologists ensured that relevant questions about oral health were included in the topic guide, along with questions to explore the participants' perspectives and experiences. Integrating the perspectives of ethnologists helped to minimise potential biases and provided both macro and micro insights into practices that odontologists might otherwise perceive as inconsequential [19]. Involving ethnologists added particular value, as their expertise in cultural analysis, qualitative methodology and inductive coding strengthened the study's analytical depth and reflexivity, enabling a more comprehensive understanding of how organisational practices and professional cultures shape oral health care approaches and perspectives. The first author, a female ethnologist and research assistant at the time, acknowledges that her background and prior experiences may have influenced data collection, analysis and interpretation. Recognising that the researcher always interprets qualitative data, the diverse team of co‐authors, including two ethnologists and two odontologists, both men and women, with a wide range of ages and academic ranks from PhD student to Professor, collaboratively contributed perspectives during analysis, ensuring a balanced and reflexive interpretation.

### 2.2 Data Analysis

All focus group discussions were selectively transcribed and subsequently analysed with the aid of NVivo 14 [20]. The study is grounded in a pragmatic constructive paradigm, emphasising the co‐construction of meaning through interaction. In line with this, an interpretative approach is applied to data analysis, focusing on understanding participants' perspectives and the context in which they are embedded. The analysis followed Braun and Clarke's thematic approach, which emphasises researcher reflexivity and an iterative process of theme development [18]. This methodology facilitates a comprehensive engagement with the data, enabling nuanced interpretation and the identification of salient patterns within complex qualitative material. The analysis was conducted to identify emic themes and patterns emerging from participants' experiences and views, allowing the analysis to remain grounded in participants' perspectives rather than being guided by predefined theoretical constructs [18]. The procedure consists of five steps. In step 1, researchers engaged in repeated listening and reading of the transcripts in order to become thoroughly familiar with the data. Step 2 involved the generation of main codes through open coding. These were initially based on the themes from the topic guide mentioned earlier. In step 3, transcripts were read, interpreted and discussed within the broader research team. The objective of this dialogue was to reach a consensus on the main codes through critical reflection and discussion. Such interdisciplinary engagement contributed to more refined coding and fostered deeper analytical insights. In step 4, coding was performed again. This time, guided by the main codes to develop more specific subcodes for the final codebook. In the final step 5, the codebook was presented to the rest of the research team, in order to reach consensus on the interpretation and codes. Steps 1–2 and 4 were done by two ethnologists, and steps 3 and 5 were done by the entire research team, including the odontologists [20, 21].

### 2.3 Ethics and Consent

All participants provided written consent to participate and allowed their contribution to be used for research purposes. The data were stored on an encrypted secure drive at the University of Copenhagen. All participants have been pseudonymised to protect personal data. This study was approved by the Danish Data Protection Agency (514–0599/21–3000) and the local Ethical Committee at the Faculty of Health and Medical Sciences, University of Copenhagen (504–0241/21–5000).

## 3 Results

In exploring the perspectives and approach to oral healthcare between aged care and dental care services, two professional groups were included in the study: staff and managers from aged care on the one side and staff and managers from dental care on the other (Table 1). Differences in approach became evident in the data when the professions experienced miscommunication or had difficulty finding common ground, which created challenges for collaboration. It is essential to state that both parties had the best intentions for the older adults, wishing to provide the highest quality care and aiming to foster and prioritise good collaboration. For example, a dental care staff member said, ‘Aged care staff are essential collaborative partners, because they know the older adult well’. Likewise, a manager from aged care expressed, ‘It is nice and reassuring to feel that everyone is skilled and professional. However, we could wish for better and closer collaboration’. Divergent approaches were identified in communication, expectations and daily workflows, creating challenges in collaboration and ultimately affecting the oral health of the target group, which will be further explored in the following four result sections. These discrepancies fall under four analytic themes: (1) knowledge of oral health and oral care, (2) prioritisation of oral health and oral care, (3) care practice in relation to oral health and oral care and (4) handling refusal of oral care (Table 2).

**TABLE 2:** Overall themes related to oral health and oral care, aged care and dental care staffs' approaches and the potential conflicts between the two professions.

Themes related to Oral health and Oral care					
	Aged care		Dental care		
Theme	Approach	Specification	Approach	Specification	Conflict
Knowledge	Rudimentary	* Self‐taught by experience * Methods based on practice knowledge * No quality standards or guidelines	Profound	* Expert knowledge by formal education and training * Access to the latest knowledge and evidence‐based methods * Use of high‐quality standards and guidelines	Conflicts arise when caregivers feel belittled and undervalued, and dental care staff overlook the fact that they do not share the same level of knowledge.
Prioritisation	Holistic	* The older adult is viewed as a whole person. * Oral care finds priority among a list of many other care tasks.	Specific	* Specifically focused on oral health needs * Oral health and oral care have the highest priority.	Conflicts arise when establishing a balance that considers the particular oral health requirements and the individual's general health and quality of life while preserving the relationship.
Care practice	Rehabilitative	* Help to self‐help * Training the individual to regain lost functions	Compensatory	* Hands‐on support * Minimise the consequences of lost functions by care and support	Conflicts arise from varying opinions on the right timing and degree of hands‐on support for oral care at different stages of frailty.
Refusals	The right to self‐determination	* The individual's autonomy in making decisions regarding their own life	Duty of (oral) care	* The obligation to guarantee that individuals receive optimal care and protection	Conflicts arise when considering the appropriateness of overriding an older adult's refusal of consent to oral care.

### 3.1 Knowledge of Oral Health and Oral Care

Dental care staff clearly had profound expertise in oral health and oral care. In contrast, the findings reveal that aged care staff expressed a perceived lack of basic knowledge regarding oral health, of which they were highly conscious.

Interviewer: Are you adequately trained to care for the older adults' mouths and teeth?

Aged care staff member 1: I think I lack competence.

[The other participants agree.]

Interviewer: How much of your education covers this?

Aged care staff member 2: Nothing. Maybe just a single chapter (Aged care).

The extent of oral care focus during education varied between professional groups, with nurses having it as a distinct and mandatory part of their training. In contrast, nursing assistants expressed curiosity about the subject and viewed it as an area for potential improvement. All focus groups that included aged care staff requested further training and knowledge as illustrated by their posing oral health‐related questions and seeking the researchers' advice on matters related to oral health and oral care during the focus groups.

The lack of knowledge among aged care staff resulted in varied approaches to performing oral care, and no consistency was found in the frequency of assistance with tooth brushing. Aged care staff helped a few older adults with brushing once a day but could not explain why one individual required assistance while others did not, despite having the same level of care dependency. Others did not help older adults with oral care at all, and an aged care staff member said, ‘It is only when there is a problem with the mouth or teeth that we look inside the mouth. That's when the older adult expresses that it hurts in the mouth’. Staff followed no official procedures when carrying out oral care practice. Instead, aged care staff relied on self‐taught methods based on personal experiences, which led to varied approaches to performing oral care.

Interviewer: So, what do you draw on?

Aged care staff member 1: Personal experience

Aged care staff member 2: Yes, entirely.

Aged care staff member 1: Yes, because you've learned it best yourself

Aged care staff member 2: Many of us have children, and we've also brushed their teeth (Aged care)

Dental care staff expressed frustrations related to older adults repeatedly presenting with poor oral hygiene and showing no positive development in oral hygiene despite annotations communicated to aged care staff. The dental care had a standing educational offer to the aged care but had not received a request for it:

Every time we visit a new nursing home, we should provide the managers with pamphlets about our educational offerings for aged care staff, as it seems they are unaware of the offer. Perhaps the obstacle is that the training often takes place during working hours in an already understaffed sector. I find that the staff are curious, especially the trainees on internships. (Dental care)

The two levels of knowledge challenged the collaboration between dental care staff and aged care staff. The emerging frustration negatively affected their communication and willingness to cooperate.

### 3.2 Prioritisation of Oral Health and Oral Care

The theme of knowledge intersects with the theme of prioritisation, as dental care staff found that oral care was not prioritised if the aged care staff were unaware of the consequences of poor oral health. In general, dental care and aged care staff described divergent views on the prioritisation of oral care, and the aged care staff's holistic approach to prioritising care tasks contrasted with the dental care staff's specific focus on oral health. A dental care staff member elaborated, ‘They [aged care staff] can do many other things with demented people if they must, but they can't brush their teeth. That surprises me, I would say. And I think it's a real pity, because I can see that some of these citizens have nice teeth, but [they] decay when they come to a nursing home’. The primary concern for dental care staff was to ensure good oral health, which they regarded as a matter of professional integrity and pride. While aged care staff recognised the importance of oral care, they had other priorities beyond it. They delivered care to older adults who were dependent in various ways, and they often managed a diverse range of tasks that differed from one individual to another. Moreover, in daily care, aged care staff had to prioritise not only the specific tasks of each individual but also the needs of multiple older adults. Additionally, factors such as rigid schedules, staff substitutions and staff without formal training sometimes led to oral care being de‐prioritised. Aged care staff expressed that they occasionally faced a lack of understanding from dental care staff regarding their holistic approach to daily prioritisation. An aged care staff member elaborated on the circumstances and conditions typical to their workday:

What should I do here? Should I prioritise mouth and oral care? It might take a long time if the older adult says no. Should I prioritise bathing them? Should I prioritise feeding them? Should I prioritise giving them the information I know they need? Should I prioritise the documentation that is also part of the visit? (Aged care)

Information on the mouth and teeth was not documented in the care records, which also hindered prioritisation of oral care. An aged care staff member said, ‘In the 20 years I have worked in aged care, I have never read anything about the teeth in an older adult's record’. Unlike oral care, perineal care was highlighted as a task with high prioritisation, meticulous record keeping and official procedures. ‘What we really focus on is the older adult's bottom. [The group laughs.] The bottom and diapers take up a lot of space. We talk about it a lot, and it determines whether we do our job correctly’, said an aged care staff member.

Prioritisation was also an issue in relation to management focus. Aged care staff expressed that oral care was not highly prioritised by managers, which influenced their ability to prioritise it in daily care practice. A manager said, ‘With us, they often come with issues that aren't necessarily about oral health. The oral part tends to be overshadowed. We might actually wish for a bit more structure’.

### 3.3 Care Practice in Relation to Oral Health and Oral Care

When the aged care staff prioritised oral care, they described a general rehabilitative care approach, meaning that their mindset in everyday care practice was focused on supporting and training older adults to be independent insofar as it was safe in terms of the individual's health. This also went for their approach to oral care practice, which was more observant and less hands‐on supportive for tooth brushing. An aged care staff member elaborated, ‘We work rehabilitatively. The older adults should be able to do most things themselves’. In comparison, dental care staff advocated for a compensatory approach to oral care practice and argued for earlier hands‐on support in the initial stages of frailty. These two contrasting care approaches complicate collaboration, particularly regarding the appropriate timing of oral care support in the progression of frailty. A dental care staff member elaborated on optimal oral care: ‘Brushing teeth once or twice a day is the solution. Aged care staff do not know how to brush the teeth of older adults. When we return after six months, no brushing has been done since’.

When describing care practice, diaper and perineal hygiene were again compared to tooth brushing. Dental care staff expressed confusion about how aged care staff always managed to change the diaper, regardless of how uncooperative the older adult might be, yet struggled with tooth brushing, stating that the consequences were equally severe. An aged care staff member reflected on this comparison and their differing approach and attitudes:

It may also be about our approach, because an older adult can say no to having their diaper changed, but here you are like, ‘You have to!’ So, this is perhaps also about how you meet them yourself. There's this ‘That's just the way it is’ mentality, and probably not so much when it comes to teeth and oral care. You are more ethical about it. You would stand there and open the labia and wash in between, but you don't even think you can bring yourself to brush the inside of the mouth around the molars. There is more significant embarrassment about it. They also find oral care unpleasant, but perhaps it's more about our attitude. (Aged care)

The aged care staff member described her mindset and attitude towards perineal hygiene, an area in which she was an expert, explaining that it differed from performing oral care and articulating this with a sense of shame and a different attitude. In the same way, a dental care staff member talked about professionalism regarding different care tasks:

They [aged care staff] are experts at changing diapers. For me, this is more intimate than providing oral care. This probably has something to do with our skills and education. They have a certain attitude toward oral care. … I have experienced aged care staff leaving the room in disgust while I was working. (Dental care)

### 3.4 Handling Refusals of Oral Care

The topic of how to handle an older adult who refuses oral care often arose, and the two professions had conflicting mindsets and approaches to handling this. Aged care staff argued for older adults' right to self‐determination. An aged care manager said,

We must not violate the older adult's right to self‐determination. Is the older adult themself interested in having their mouth treated? We must have tried everything else first. And we must have documented everything before we can proceed [with using force]. It's like when people are about to step onto the road. So, it's only right before the car hits them that we remove them. (Aged care)

In contrast, dental care staff argued that neglecting to perform oral care constituted a failure in care responsibilities, in the words of one participant: ‘When we see that the older adult hasn't had their teeth brushed for a long time, I feel sorry for them and think it's beneath their dignity. It is for the older adult's sake that their teeth should be brushed’ (Dental care). Both parties aimed to uphold the dignity of older adults, but they had different views on how to achieve it. Dental care staff believed that leaving older adults with inadequate oral hygiene was undignified. Conversely, aged care staff argued that respecting older adults' refusal of oral care was a matter of dignity.

The aged care staff's solution to older adults refusing oral care involved cultivating the relationship between aged care staff and the older adult, aiming to obtain permission to perform oral care. ‘We always put the relationship before any care task’ (Aged care). The aged care staff considered this preferable in terms of not damaging the good relationship between them and the older adult. The primary tool was pedagogical motivational work that aimed to help the older adults understand the consequences of poor oral hygiene. This posed an articulation challenge for aged care staff, as they lacked knowledge about the consequences. Another strategy was to enlist the help of a colleague with whom the older adult already had a good relationship. At times, it was helpful to pretend to be a dental care staff member, as they were perceived as having more authority, thereby facilitating easier access to the mouth. A third strategy involved returning to the older adults at another time, but this often resulted in aged care staff forgetting to return to perform oral care.

Aged care staff often suspected that the older adults were lying about performing oral care on their own and found it difficult to persuade them to open their mouths. This mistrust could impact the relationship, which was more crucial for aged care staff to maintain than any caregiving task. ‘They must not feel treated like children. If they say they have done it and we want to help them afterwards, it's as if we don't believe them’, an aged care staff member said.

A challenge specifically expressed by aged care staff in home care was working in older adults' homes. The domestic setting emphasised that the older adult oversaw decisions, strengthening their right to self‐determination in this private sphere. This made it more difficult for aged care staff to perform tasks that older adults resisted, including oral care. While older adults also reside in a private setting in nursing homes, this was not described as a challenge to the same extent.

## 4 Discussion

This study provides new insights into the organisational and interpersonal factors that facilitate or hinder collaboration between aged care and dental care staff, highlighting the contextual challenges and differing professional priorities that shape care practices. By questioning assumptions about how interdisciplinary care is conceptualised and prioritised, this work advances understanding of why oral health often remains marginalised in primary care for older adults. Through the interpretive process of the inductive analysis, this study identified four divergent approaches to oral health and oral care between aged care and dental care staff. Dental care staff possess profound knowledge of oral health and oral care, in contrast to aged care staff, who possess rudimentary knowledge. Dental care staff also focus specifically on oral health, whereas aged care staff take a more holistic approach to the individual. Further, aged care staff described a rehabilitative practice to oral care as compared to dental care staff, who spoke for more and earlier hands‐on support. Moreover, aged care staff regarded the right to self‐determination as crucial, contradicting dental care staff's adherence to the duty of care in situations in which the older adult refuses oral care.

In a Scandinavian context, studies highlight that organisational boundaries, unclear responsibilities, and differences in professional priorities often challenge collaboration between aged care staff and dental care staff. Research from Norway and Sweden shows that care staff generally express positive attitudes towards oral health but experience limited time, training, and support from dental professionals [22, 23, 24]. The mobile or outreach dental services are valued for improving access and providing guidance, yet communication and coordination between services remain inconsistent. Scandinavian studies were chosen for comparison because their organisation of publicly funded dental care for older adults most closely resembles the Danish model. These findings suggest that while the Scandinavian system provides a favourable structural framework, successful implementation of interdisciplinary oral care still depends on local collaboration practices, shared understanding of roles and mutual professional respect between aged care and dental care staff.

The rudimentary knowledge and lack of skills and training on oral health and oral care among aged care staff are commonly mentioned obstacles to oral care [25, 26, 27, 28]. This issue is evident not only for aged care staff but also for a wide range of care providers working in different non‐dental sectors [29], indicating a general knowledge problem on oral health outside dentistry. In this regard, training programmes, education and practical skills have been shown to enhance knowledge and raise awareness of oral health in aged care staff, but they have not resulted in lasting change [30, 31, 32, 33]. This indicates that improvement of knowledge among aged care staff cannot stand alone as an intervention to improve oral health in care‐dependent older adults.

Other studies have also identified aged care staff's holistic view on prioritising care tasks and the resulting difficulties in prioritising oral care as barriers to providing oral health care [34, 35, 36]. Naturally, this can provoke dental care staff, as it can be seen as de‐prioritising oral health. This may be the case but might also (as described in this study) simply be a matter of having to juggle multiple care tasks in a limited time. Whatever the case, an understanding of each other's reasons for prioritisation is needed to avoid blame and improve collaboration between aged care and dental care. In this regard, Folker et al. introduce the concept of tooth shame and argue that oral health problems can elicit distinct and profound feelings of shame in older adults and aged care staff involved in performing oral care [37]. This implies that oral care is not prioritised due to such shame‐related responses. This can challenge collaboration between aged care and dental care, as dental care staff have difficulties in understanding this shame [37]. However, it is important to understand that accusing aged care staff of ignorance and neglect would further shame them, resulting in even more difficulties in prioritising oral care [37].

No prior studies have explored the discrepancy in general care practice between aged care and dental care. We believe, however, that our findings on divergent care practices are meaningful. Since the beginning of year 2000, Denmark and several other welfare states have implemented a series of reforms aimed at rendering aged care rehabilitative and fostering the independence of older adults [38, 39], with the care approach shifting from a compensatory practice to a rehabilitative one. Where aged care was previously perceived as ‘dirty work’ because aged care staff had to take care of older adults' bodies, it is now increasingly framed as coaching and motivating rather than directly service oriented when older adults are expected to handle these tasks themselves through training [38]. This shift positively contributes to aged care staff's identity [38]. It seems, however, that the shift is not adopted by dental care staff, who every day see mouths in need of better hygiene measures. They naturally argue for earlier hands‐on support but face a competing practice [6]. Again, this is important to know to ensure a good collaboration between the two professions.

Aged care staff have reported that older adults' verbally declining or refusing warranted aid for oral care constitutes a barrier to performing oral care as described in our study [40, 41]. Because aged care staff consider refusal as the older adult's right, the only solution for them is to work on the relationship with the older adult and hope that help will be accepted at a later stage. The Danish legislation is somewhat unclear in this regard. Aged care staff are obliged to provide care if needed, and force can even be used to provide that care [42], but force should be a last resort taken only after rigorous motivation and should be considered in light of the care task and the consequences of not providing care [42]. The approach is thus open to interpretation, and aged care staff members choose motivation, whereas dental care professionals do not rule out the use of force. It seems, however, that dental care staff have a key to accessing older adults' mouths, as aged care staff mention pretending to be dental care staff to gain access. Interdisciplinary collaboration is obviously needed. Aged care staff know the older adult and can help dental care staff have a good relationship with them, whereas dental care staff can help aged care staff gain access to oral care.

As mentioned, numerous studies and the international consensus argue that interdisciplinary collaboration facilitates enhancing oral health and oral care for care‐dependent older adults [43, 44]. Nonetheless, this study shows that collaboration between aged care and dental care can be difficult as noted in several other studies [11, 45]. Limited experience within healthcare teams and a lack of awareness of oral health are reported barriers that hinder dental care staff from contributing effectively to an interdisciplinary team in aged care [11]. Other obstacles to interdisciplinary collaboration in aged care are issues related to team performance, organisational structures and information sharing [45].

A limitation of this study is that participants were all recruited from the Capital Region of Denmark, which may have influenced the findings by reflecting geographical‐specific organisational cultures and practices. Although the study included a relatively large number of focus groups, the strength of the data lies in its richness and depth rather than quantity. Future research should therefore explore similar dynamics across a broader range of rural and urban settings to enhance transferability. Although there were variations in training, knowledge and organisational structures across professional groups and settings (home care vs. nursing homes) within aged care, we deliberately chose not to differentiate the findings. This decision was based on the analysis that similar approaches and challenges were reported across the broader group of aged care staff and settings. By focusing on overarching themes, we aimed to capture the collective experiences and practices within aged care, while acknowledging that minor intra‐group differences may exist.

Additionally, member validation was not conducted, although the analysis was inductive and interpretive. Instead, the findings were discussed within the interdisciplinary research team to reach consensus and ensure a shared interpretation of the data. Another possible limitation is that some participants might have felt less comfortable speaking openly in mixed groups (e.g., aged care managers and dental care managers). However, participants appeared to speak freely, and aged care managers were the majority in each group, which may have facilitated openness by having like‐minded colleagues in the group. Although focus groups offered meaningful discussions and diverse viewpoints, the data could have benefited from the use of additional methodologies. Conducting individual interviews may have revealed personal and emotional insights in more detail, providing a deeper understanding of the participants' experiences. Furthermore, the inclusion of questionnaires could have facilitated further data triangulation, leading to a more thorough analysis of the collaboration. The transferability of the findings may be limited due to the study's Danish context within a welfare state framework; however, the approaches to dental care employed by the various professional groups are likely to be globally recognised.

The study's findings can benefit multiple stakeholders. Healthcare professionals, particularly those in aged care and dental care, can use the insights to improve interdisciplinary collaboration and address and prevent conflicts arising in oral care for older adults. Additionally, educators and researchers in health science disciplines can integrate the results into curricula and further investigations, fostering a more comprehensive understanding of collaborative oral care dynamics. Further research is required to explore how dental care staff can become a more integrated part of an interdisciplinary health team in the aged care sector. A co‐creation approach involving representatives from both professionals and managers would be a beneficial method.

## 5 Conclusion

This study offers a deep understanding of the divergent approaches to oral care in aged care and dental care and their potential impact on collaboration. Coexisting approaches to oral care were identified, which pose a challenge to collaboration and, consequently, to the oral health of care‐dependent older adults. The findings highlight the complexities of interdisciplinary collaboration. Building a shared approach to oral care on the basis of this study's findings could improve the oral health of care‐dependent older adults.

## Author Contributions

All authors provided substantial contributions to the conception, design, data collection and analysis. L.E.A. specifically drafted this manuscript and E.B.Ø. contributed significantly to the manuscript, providing substantial input and revisions. All authors have read and approved the final manuscript for submission.

## Funding

This work was supported by Velux Fonden (00040212). Sygeforsikring ‘danmark’ (2020‐0343).

## Conflicts of Interest

The authors declare no conflicts of interest.

## Supporting information


**Data S1:** COREQ (COnsolidated criteria for REporting Qualitative research) Checklist

## Data Availability

Research data are not shared.
